# Prevalence and Correlates of Clinical Nephropathy in Patients With Type 2 Diabetes at Abia State Specialist Hospital and Diagnostic Center, Umuahia, Nigeria: A Cross-Sectional Study

**DOI:** 10.7759/cureus.86962

**Published:** 2025-06-29

**Authors:** Izuchukwu E Okeji, Onyedikachi F Uzor, Christopher C Okafor, Silas U Okafor, Augustine I Airaodion, Obinna J Orji, Isaiah O Abali, Ikpembhosa J Esangbedo, Oladoyin Ogunbayo Jolaoye, Temitayo O Olaotan, Innocent Chima Zacs, Akudo B Umeh, Ugonna Emmanuella Ojumonu, Abasiekeme Monday Ekwere, Cynthia Kenechukwu Madueke, Ochuko Austin-Jemifor, Excel Nwasinachi Victor-Anozie, Augustine C Amuta, Chibuike Umeh

**Affiliations:** 1 General Medicine, North Cumbria Integrated Care, NHS Foundation Trust, North Cumbria, GBR; 2 General Practice, General Practice/Forge Health Sheffield Teaching Hospital, Sheffield, GBR; 3 Internal Medicine, Ebonyi State University Teaching Hospital, Abakaliki, NGA; 4 Internal Medicine, Abia State Specialist Hospital and Diagnostic Center, Umuahia, NGA; 5 Emergency Medicine, Maimonides Medical Center, Brooklyn, USA; 6 Biochemistry, Federal University of Technology, Owerri, NGA; 7 Biochemistry, Lead City University, Ibadan, NGA; 8 Acute Medicine, University Hospital of Derby and Burton, NHS Foundation Trust, Derby, GBR; 9 Surgery, Abia State University Teaching Hospital, Aba, NGA; 10 Internal Medicine, University College Hospital, Ibadan, NGA; 11 Internal Medicine-Pediatrics, University of Illinois College of Medicine Peoria, Peoria, USA; 12 Internal Medicine-Pediatrics, OSF Medical Center, Peoria, USA; 13 Acute Medicine, Wythenshawe Hospital, Manchester University, NHS Foundation Trust, Manchester, GBR; 14 Internal Medicine, Abia State University Teaching Hospital, Aba, NGA; 15 General Medicine, Latham House Medical Practice, Melton Mowbray, Leicestershire, GBR; 16 Internal Medicine, Federal Medical Center, Umuahia, NGA; 17 Internal Medicine, All Saints University, Roseau, DMA; 18 Internal Medicine, American University of Barbados, Bridgetown, BRB; 19 Internal Medicine, Queen Elizabeth Hospital Birmingham, University Hospitals Birmingham, NHS Foundation Trust, Birmingham, GBR; 20 Internal Medicine, St. Joseph Catholic Hospital, Asaba, NGA; 21 Health and Wellness Division, Prince George’s County Health Department, Largo, USA; 22 Emergency Medicine, University Hospitals of Leicester, NHS Foundation Trust, Leicester, GBR

**Keywords:** albuminuria, clinical nephropathy, kidney disease, t2dm, type 2 diabetes mellitus

## Abstract

Background

Clinical nephropathy is a prevalent and serious microvascular complication of type 2 diabetes mellitus (T2DM), contributing substantially to increased morbidity and mortality among affected individuals. It often progresses insidiously, leading to end-stage renal disease if not promptly detected and managed. Despite its growing public health importance, there is a paucity of region-specific data on the prevalence and correlates of clinical nephropathy in patients with T2DM, particularly in resource-limited settings. In regions such as Abia State, Nigeria, limited research has been conducted to determine the burden of diabetic nephropathy and its clinical correlates. Understanding the local epidemiology of this complication is essential for guiding early detection strategies, optimizing management, and improving patient outcomes.

Objective

This study investigates the prevalence and correlates of clinical nephropathy among patients with type 2 diabetes at Abia State Specialist Hospital and Diagnostic Center, Nigeria, to inform strategies for improving early detection and management of diabetic nephropathy complications.

Methodology

A cross-sectional study design was employed at the Abia State Specialist Hospital and Diagnostic Center outpatient clinic. The sample size (*n* = 255) was calculated using the Cochran formula, considering a known prevalence of nephropathy in patients with T2DM in Nigeria. Data were collected through structured interviews, clinical examinations, and laboratory procedures, and analyzed using IBM SPSS Statistics for Windows, Version 20 (IBM Corp., Armonk, NY).

Results

Clinical information indicated a high prevalence of clinical nephropathy (77, 30.20%). Lifestyle factors reflected low tobacco use (25, 9.80%) and alcohol consumption (32, 12.55%), with frequent engagement in physical activity (87, 34.12%). Access to healthcare revealed barriers (174, 68.24%) and limited knowledge of diabetic kidney disease (77, 30.20%). Anthropometric indices and blood pressure showed significant differences between groups (*P* < 0.05), with diabetic patients with nephropathy exhibiting higher values. Renal indices also displayed significant differences (*P* < 0.05), indicating renal dysfunction among diabetic patients with nephropathy. The prevalence of albuminuria was notable (microalbuminuria: 156, 61.18%). The staging of clinical nephropathy revealed varying severity levels.

Conclusions

The clinical nephropathy prevalence among patients with T2DM at Abia State Specialist Hospital and Diagnostic Center, Umuahia, Nigeria, is substantial. Factors such as anthropometric indices, blood pressure, renal parameters, glycemic control, and lipid profiles significantly correlate with the presence of nephropathy. These findings underscore the importance of early detection and management strategies to mitigate diabetic nephropathy complications

## Introduction

Diabetes mellitus, a chronic metabolic disorder characterized by hyperglycemia, represents a significant public health concern globally [1]. Type 2 diabetes mellitus (T2DM) is the most prevalent form, accounting for approximately 90% of all cases worldwide. One of the most serious complications of T2DM is diabetic nephropathy, a progressive kidney disease that can lead to end-stage renal disease (ESRD) if left untreated [2]. Diabetic nephropathy is a leading cause of morbidity and mortality among individuals with T2DM, imposing a considerable burden on healthcare systems and economies worldwide [2]. While diabetic nephropathy has been extensively studied in various populations, data remain scarce regarding its prevalence and correlates among patients with T2DM in Abia State, Nigeria.

Nigeria, the most populous country in Africa, is experiencing a rapid epidemiological transition characterized by a rising prevalence of non-communicable diseases (NCDs), including T2DM. According to recent estimates, Nigeria has one of the highest burdens of diabetes in sub-Saharan Africa, with approximately 5 million individuals living with the disease [3]. Additionally, studies have suggested that diabetic nephropathy is a significant contributor to the burden of kidney disease in Nigeria [4]. However, there is limited research specifically examining the prevalence and correlates of clinical nephropathy among patients with T2DM in Abia State, a densely populated state located in southeastern Nigeria.

Abia State presents a unique setting for studying the prevalence and correlates of diabetic nephropathy due to its diverse population, socio-economic characteristics, healthcare infrastructure challenges, and rural environment. Moreover, the prevalence of T2DM is believed to be increasing in this region, driven by factors such as urbanization, sedentary lifestyles, and changes in dietary patterns. Despite these trends, there is a paucity of data on the burden of diabetic nephropathy and its associated risk factors in Abia State.

Understanding the prevalence and correlates of clinical nephropathy in patients with T2DM in Abia State is crucial for several reasons. First, it can provide valuable insights into the magnitude of the problem and help healthcare providers allocate resources effectively to prevent and manage diabetic nephropathy. Second, identifying the correlates of nephropathy can inform targeted interventions aimed at reducing the risk of kidney complications among individuals with T2DM in this region. Additionally, studying the prevalence of diabetic nephropathy in Abia State can contribute to the existing literature on the epidemiology of diabetes and its complications in sub-Saharan Africa.

The primary objective of this research is to determine the prevalence of clinical nephropathy among patients with T2DM in the Abia State Specialist Hospital and Diagnostic Center outpatient clinic, Umuahia, Nigeria. In addition, the study aims to identify the correlates of diabetic nephropathy in this population, including demographic, clinical, and socioeconomic factors.

By highlighting the prevalence and correlates of diabetic nephropathy, the study aims to provide insights into the need for early detection and management strategies to reduce the burden of kidney complications among individuals with T2DM. Such efforts are expected to improve patient outcomes and lessen the healthcare impact of diabetic nephropathy in Abia State.

## Materials and methods

Study design

This research adopts a cross-sectional study design to investigate the prevalence and correlates of clinical nephropathy in patients with T2DM at Abia State Specialist Hospital and Diagnostic Center, Umuahia, Nigeria. Cross-sectional studies are ideal for assessing prevalence and associations between variables at a single point in time [5].

Study setting

The study was carried out at the Abia State Specialist Hospital and Diagnostic Center outpatient clinic between April and November 2024.

Study participants and eligibility criteria

The study population comprised adult patients diagnosed with T2DM who attended the outpatient clinic of Abia State Specialist Hospital and Diagnostic Center during the study period.

Inclusion criteria

The study population comprised adult patients aged 18 years and above with a confirmed diagnosis of T2DM for at least one year, who attended the outpatient clinic of Abia State Specialist Hospital and Diagnostic Center during the study period. Only patients who were able to provide informed consent and had relevant clinical and laboratory data available were included. Participants with urine albumin-to-creatinine ratio (ACR) values ranging from 30 to 300 mg/g, consistent with microalbuminuria, and/or ACR values above 300 mg/g, consistent with macroalbuminuria, indicative of nephropathy, were included.

Exclusion criteria

Patients were excluded if they had type 1 diabetes mellitus, were pregnant, had known chronic kidney disease from non-diabetic causes, or were critically ill or mentally incapacitated and unable to participate.

Sample size determination

The sample size for this study was calculated using the Cochran formula for estimating proportions in a population, as outlined by Ijioma et al. [6]. The formula used was

$n = \frac{Z^2 (Pq)}{e^2}\text{}$

where *n* represented the minimum sample size, *Z* was the *Z*-score at a 95% confidence level (1.96), *P* was the known prevalence of diabetic nephropathy among patients with T2DM in Nigeria, *e* was the margin of error tolerated at 5% (0.05), and *q* was equal to 1 - *P*.

According to Rafiu et al., the existing prevalence of nephropathy in patients with T2DM in Nigeria is 18.3% (*P* = 0.183) [7], making *q* equal to 0.817. 

Substituting these values into the formula, the calculation was

$n = \frac{1.96^2 (0.183 * 0.817)}{0.05^2}\text{}$

resulting in a minimum sample size of 229.74, which was rounded up to 230. To account for a potential non-response rate of 10%, the sample size was further adjusted to 255.

Sampling technique

A systematic random sampling technique was employed to avoid selection bias. The sampling frame consisted of the list of patients with T2DM attending the outpatient clinic at the Abia State Specialist Hospital and Diagnostic Center during the study period. One out of every two patients who met the inclusion criteria was selected until the sample size was achieved during the clinic days.

Data collection instrument

A structured questionnaire developed by the authors was used to collect data, and the questionnaire was developed from various studies (Appendix) [8-24]. It was pre-tested on a sample of 25 patients from a different hospital to assess its validity and reliability. Cronbach's alpha was used to assess the internal consistency of each section of the questionnaire. The clinical information section had a Cronbach's alpha of 0.72, the lifestyle and behavioral factors section had 0.75, and the access to healthcare and knowledge section had 0.78, indicating acceptable to good reliability across the components. The questionnaire covered the sociodemographic details, clinical information, lifestyle factors, and access to healthcare. Clinical examinations were conducted by trained healthcare personnel.

Determination of anthropometric indices and blood pressure

The body mass index for each participant was calculated from weight and height measurements obtained using Hanson’s weighing scale (capacity of 120 kg) and a meter rule attached to a wooden pole, respectively, as described by Agu et al. [25]. Briefly, the participants were weighed in light clothing, and the reading was taken to the nearest 0.1 kg. Height was measured to the nearest 0.1 cm with the participants standing erect on a flat surface. The weighing scale was calibrated to zero before taking the weight measurements. Calibration checks were performed daily using standard weights to verify precision and consistency throughout the data collection period.

Blood pressure (BP) measurements were taken using an Omron automatic sphygmomanometer (M2: HEM-7121-E, Vietnam). To ensure reliability, the device was regularly checked and calibrated according to the manufacturer’s guidelines before and during the study. BP was measured twice for each participant, with at least a three-minute interval between readings. Measurements were conducted by trained research assistants following standardized procedures: the participant was seated comfortably with the arm supported at heart level, as described by Ijioma et al. [26]. To minimize measurement variability, research assistants underwent training sessions and periodic assessments to establish and maintain inter-rater reliability. Inter-rater agreement was evaluated during pilot testing using the intraclass correlation coefficient (ICC), achieving a satisfactory reliability coefficient of 0.85, indicating high consistency between raters.

Laboratory procedures

Five milliliters of blood were collected from all participants. A fully automatic chemistry analyzer was used to estimate urea, creatinine, triglycerides, total cholesterol, and serum high-density lipoprotein (HDL) cholesterol. Low-density lipoprotein (LDL), very-low-density lipoprotein (VLDL), and LDL/HDL ratios were subsequently calculated. The estimated glomerular filtration rate (eGFR) was determined based on serum creatinine using the Modification of Diet in Renal Disease (MDRD) equation [27].

Capillary fasting blood glucose was measured using an Accu-Chek Active Glucometer, which reports glucose levels in mmol/L. A fasting blood glucose value of up to 7.2 mmol/L was considered within the target range for individuals with diabetes, while values above 7.2 mmol/L were considered high, by American Diabetes Association (ADA) recommendations [28].

Urine samples were tested for microalbuminuria using Microalbumin 2-1 combo strips (CLIA waived, Inc. 2-1 Creatinine/Microalbumin Rapid Test Strips), according to the manufacturer's instructions. The albumin and creatinine values obtained were used to calculate the ACR. Microalbuminuria was defined as an ACR between 30 and 300 mg/g, while values above 300 mg/g were considered indicative of macroalbuminuria [28].

Data analysis

Data were analyzed using IBM SPSS Statistics for Windows, Version 20 (IBM Corp., Armonk, NY). Descriptive statistics (frequencies, percentages, means, and standard deviations) were used to summarize the demographic and clinical characteristics of the participants. An independent t-test was used to determine whether there was a statistically significant difference in the means of continuous variables between patients with T2DM with and without nephropathy, including anthropometric indices, BP, renal indices, and lipid profile. A *P*-value of <0.05 was considered statistically significant.

Ethical approval and consent to participate

Ethical approval for the study was obtained from the Ethics Committee of Abia State Specialist and Diagnostic Hospital, Umuahia, Nigeria, with approval number ABSHDC/EC/24/039, dated March 19, 2024. Written informed consent was obtained from all participants before their enrollment in the study. Participant confidentiality was ensured by assigning identification codes in place of names, and the data was stored securely. Participation was entirely voluntary, and participants retained the right to withdraw from the study at any time without any consequences.

## Results

The sample included 255 participants. The age distribution showed that the largest proportion was aged 40-49 years, accounting for 86 participants (33.73%). This was followed by 74 participants (29.02%) aged 50-59 years and 56 participants (21.96%) aged 60 years. In terms of sex, there were more females than males in the study. Female participants comprised 163 (63.92%), while male participants accounted for 92 (36.08%). Regarding educational attainment, the majority of the participants had secondary education, with 109 individuals (42.75%). This was followed by 68 participants (26.67%) with tertiary education and 54 (21.18%) with primary education. For marital status, most participants were married, comprising 200 individuals (78.43%). Divorced or widowed participants numbered 41 (16.08%), while single participants were the fewest at 14 (5.49%), as shown in Table 1.

**Table 1:** Sociodemographic details of respondents.

Variable	Frequency (*n* = 255)	Percentage (%)
Age (in years)		
Below 20	2	0.78
20-29	19	7.45
30-39	18	7.06
40-49	86	33.73
50-59	74	29.02
60 and above	56	21.96
Sex		
Male	92	36.08
Female	163	63.92
Educational level		
No formal education	24	9.41
Primary education	54	21.18
Secondary education	109	42.75
Tertiary education	68	26.67
Marital status		
Single	14	5.49
Married	200	78.43
Divorced/Widowed	41	16.08

From Table 2, among the 255 participants, the duration of T2DM diagnosis varied: 15 (5.88%) had been diagnosed for less than one year, 31 (12.16%) for one to three years, 28 (10.98%) for three to five years, 116 (45.49%) for five to 10 years, and 65 (25.49%) for more than 10 years. All participants (255, 100.00%) reported currently receiving diabetes treatment. Regarding the type of treatment received (305 responses due to multiple selections), 138 (45.25%) were on a diet and exercise regimen, 86 (28.20%) used insulin injections, 59 (19.34%) were on oral medications, and 22 (7.21%) reported other forms of treatment. A total of 77 participants (30.20%) had been diagnosed with clinical nephropathy, while 178 (69.80%) had not. Among those with nephropathy, 27 (10.59%) had been diagnosed for less than one year, 13 (5.10%) for one to three years, 17 (6.67%) for three to five years, 9 (3.53%) for five to 10 years, and 11 (4.31%) for more than 10 years. All 77 participants (30.20%) with nephropathy were receiving treatment. When asked about undergoing dialysis or a kidney transplant, 14 participants (5.49%) responded yes. Regarding comorbid conditions, 194 participants (54.49%) reported having hypertension, 104 (29.21%) had obesity, 27 (7.58%) had heart disease, 12 (3.37%) had other chronic conditions, and 19 (5.34%) indicated no additional conditions. A significant number of participants, 212 (83.14%), had a family history of diabetes among immediate relatives (parents or siblings).

**Table 2:** Clinical information of respondents. *Multiple responses.

Variable	Frequency (*n* = 255)	Percentage (%)
How long have you been diagnosed with type 2 diabetes?		
Less than 1 year	15	5.88
1-3 years	31	12.16
3-5 years	28	10.98
5-10 years	116	45.49
More than 10 years	65	25.49
Are you currently receiving diabetes treatment?		
Yes	255	100.00
No	0	0.00
If yes, what type of treatment are you receiving for type 2 diabetes?* (Select all that apply) (*n* = 305)		
Oral medications	59	19.34
Insulin injections	86	28.20
Diet and exercise regimen	138	45.25
Others	22	7.21
Have you been diagnosed with clinical nephropathy (kidney disease)?		
Yes	77	30.20
No	178	69.80
If yes, how long have you had clinical nephropathy?		
Less than 1 year	27	10.59
1-3 years	13	5.10
3-5 years	17	6.67
5-10 years	9	3.53
More than 10 years	11	4.31
Are you currently receiving treatment for clinical nephropathy?		
Yes	77	30.20
No	0	0.00
Not applicable	178	69.80
Have you ever undergone dialysis or a kidney transplant?		
Yes	14	5.49
No	241	94.51
Do you have any other chronic health conditions?* (Select all that apply) (*n* = 356)		
Hypertension	194	54.49
Heart disease	27	7.58
Obesity	104	29.21
Others	12	3.37
Not applicable	19	5.34
Do any of your immediate family members (parents, siblings) have diabetes?		
Yes	212	83.14
No	43	16.86

Out of the 255 participants, 25 (9.80%) reported smoking tobacco products, whereas 32 participants (12.55%) consumed alcohol regularly. In terms of physical activity frequency, 87 participants (34.12%) reported always engaging in physical activity, 106 (41.57%) often did so, 34 (13.33%) sometimes exercised, and 28 (10.98%) rarely exercised. When asked about the intensity of their physical activity, 29 (11.37%) described their lifestyle as sedentary, 184 (72.16%) engaged in light activity such as walking, and 42 (16.47%) participated in moderate activities like jogging or swimming. Regarding dietary habits, 29 participants (11.37%) followed a high-fat diet, 44 (17.25%) consumed a high-carbohydrate diet, 175 (68.63%) reported having a balanced diet, and 7 (2.75%) indicated other dietary patterns, as shown in Table 3.

**Table 3:** Lifestyle and behavioral factors.

Variable	Frequency (*n* = 255)	Percentage (%)
Do you smoke tobacco products?		
Yes	25	9.80
No	230	92.16
Do you consume alcohol regularly?		
Yes	32	12.55
No	223	87.45
How often do you engage in physical activity (e.g., exercise, walking)?		
Always	87	34.12
Often	106	41.57
Sometimes	34	13.33
Rarely	28	10.98
Never	0	0.00
What is your level of physical activity? (Select one)		
Sedentary (little to no physical activity)	29	11.37
Light activity (e.g., walking)	184	72.16
Moderate activity (e.g., jogging, swimming)	42	16.47
Vigorous activity (e.g., heavy weightlifting, intense sports)	0	0.00
What is your typical dietary pattern?		
High fat	29	11.37
High carbohydrate	44	17.25
Balanced	175	68.63
Others	7	2.75

Among the 255 participants, 34 (13.33%) reported visiting a healthcare provider regularly (at least once a month) for diabetes management, 134 (52.55%) visited occasionally (every 3-6 months), and 87 (34.12%) visited rarely (once a year or less). A significant number, 174 participants (68.24%), reported encountering barriers to accessing healthcare services such as distance or cost. Regarding awareness of diabetic kidney disease, 24 participants (9.41%) considered themselves very knowledgeable, 71 (27.84%) were somewhat knowledgeable, 77 (30.20%) were not very knowledgeable, and 83 (32.55%) reported not being knowledgeable at all. Only 41 participants (16.08%) had received formal education or counseling on diabetic kidney disease, as shown in Table 4.

**Table 4:** Access to healthcare.

Variable	Frequency (*n* = 255)	Percentage (%)
How often do you visit a healthcare provider for diabetes management?		
Regularly (at least once a month)	34	13.33
Occasionally (every 3-6 months)	134	52.55
Rarely (once a year or less)	87	34.12
Have you encountered any barriers to accessing healthcare services (e.g., distance, cost)?		
Yes	174	68.24
No	81	31.76
How knowledgeable do you consider yourself about diabetic kidney disease?		
Very knowledgeable	24	9.41
Somewhat knowledgeable	71	27.84
Not very knowledgeable	77	30.20
Not knowledgeable at all	83	32.55
Have you received any formal education or counselling regarding diabetic kidney disease?		
Yes	41	16.08
No	214	83.92

As shown in Table 5, participants with nephropathy had a slightly higher average height (179.22 ± 18.43 cm) compared to those without nephropathy (173.15 ± 20.11 cm), but the difference was not statistically significant (*P* = 0.075). Similarly, the average weight was higher in the nephropathy group (59.59 ± 11.64 kg) than in the non-nephropathy group (57.33 ± 14.08 kg), though this difference also did not reach statistical significance (*P* = 0.058). However, the BMI was significantly higher among participants with nephropathy (23.16 ± 6.14 kg/m²) compared to those without (20.34 ± 4.88 kg/m²), with a statistically significant difference (*P* = 0.049). In terms of BP, both systolic and diastolic readings were significantly elevated in the nephropathy group. Participants with nephropathy had a mean systolic BP of 138.34 ± 11.32 mmHg versus 131.45 ± 4.63 mmHg in those without (*P* = 0.001) and a mean diastolic pressure of 85.18 ± 8.82 mmHg compared to 78.89 ± 7.57 mmHg in the non-nephropathy group (*P* = 0.002).

**Table 5:** Anthropometric indices and blood pressure of patients with type 2 diabetes. Results are presented as mean ± standard deviation. *t* = independent t-test statistics used to compare the means between groups; *P* < 0.05 indicates statistical significance.

Anthropometric indices	With nephropathy	Without nephropathy	t	*P*-value
Height (cm)	179.22 ± 18.43	173.15 ± 20.11	3.14	0.075
Weight (kg)	59.59 ± 11.64	57.33 ± 14.08	3.78	0.058
Body mass index (BMI) (kg/m^2^)	23.16 ± 6.14	20.34 ± 4.88	3.91	0.049
Systolic blood pressure (mmHg)	138.34 ± 11.32	131.45 ± 4.63	8.67	0.081
Diastolic blood pressure (mmHg)	85.18 ± 8.82	78.89 ± 7.57	7.62	0.022

The study further showed that respondents with nephropathy show higher values for urea (30.27 mg/dL), creatinine (1.51 mg/dL), uric acid (9.13 mg/dL), and lower estimated glomerular filtration rate (eGFR) (43.38 mL/minute) when compared to those without nephropathy (Table 6).

**Table 6:** Renal indices of patients with diabetes. Results are presented as mean ± standard deviation. *t* = independent t-test statistics used to compare the means between groups; *P* < 0.05 indicates statistical significance.

Renal parameters	With nephropathy	Without nephropathy	t	*P*-value
Urea (mg/dL)	30.27 ± 3.82	19.02 ± 3.22	9.84	0.000
Creatinine (mg/dL)	1.51 ± 0.05	0.97 ± 0.01	8.95	0.010
Uric acid (mg/dL)	9.13 ± 1.01	5.26 ± 0.82	9.21	0.009
eGFR (mL/minute)	43.38 ± 6.34	99.06 ± 17.74	9.43	0.000

Table 7 indicates a higher prevalence of microalbuminuria (156, 61.18%) among patients with diabetes.

**Table 7:** Prevalence of albuminuria in patients with diabetes.

Urine albumin levels	Frequency (*n* = 255)	Percentage (%)
Normal albuminuria	88	34.51
Microalbuminuria	156	61.18
Macroalbuminuria	11	4.31

**Figure 1:**
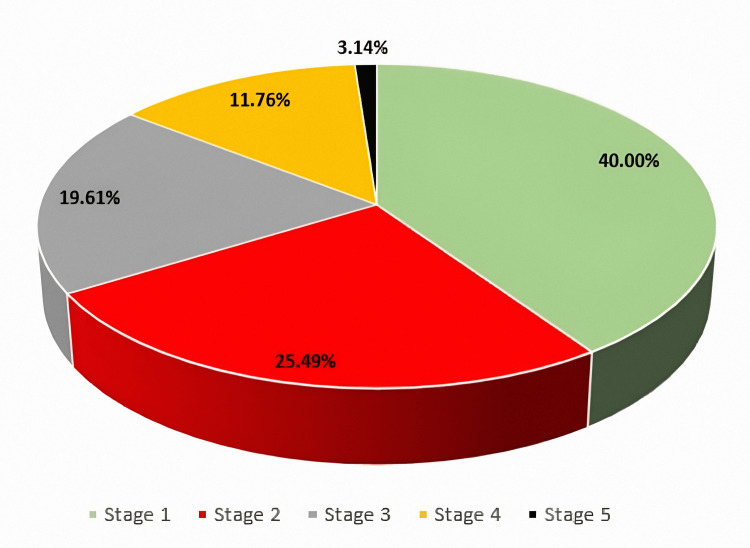
Stages of clinical nephropathy. Stage 1: Kidney damage with normal or increased GFR (>90 mL/minute). Stage 2: Mild decrease in GFR (60-89 mL/minute). Stage 3: Moderate decrease in GFR (30-59 mL/minute). Stage 4: Severe decrease in GFR (15-29 mL/minute). Stage 5: Kidney failure (<15 mL/minute). GFR, glomerular filtration rate

The findings presented in Figure 1 revealed that many participants (102, 40.00%) are in stage 1 of clinical nephropathy, while 65 (25.49%) are in stage 2, 50 (19.61%) are in stage 3, 30 (11.76%) are in stage 4, and only a few (8, 3.14%) are in stage 5.

**Figure 2:**
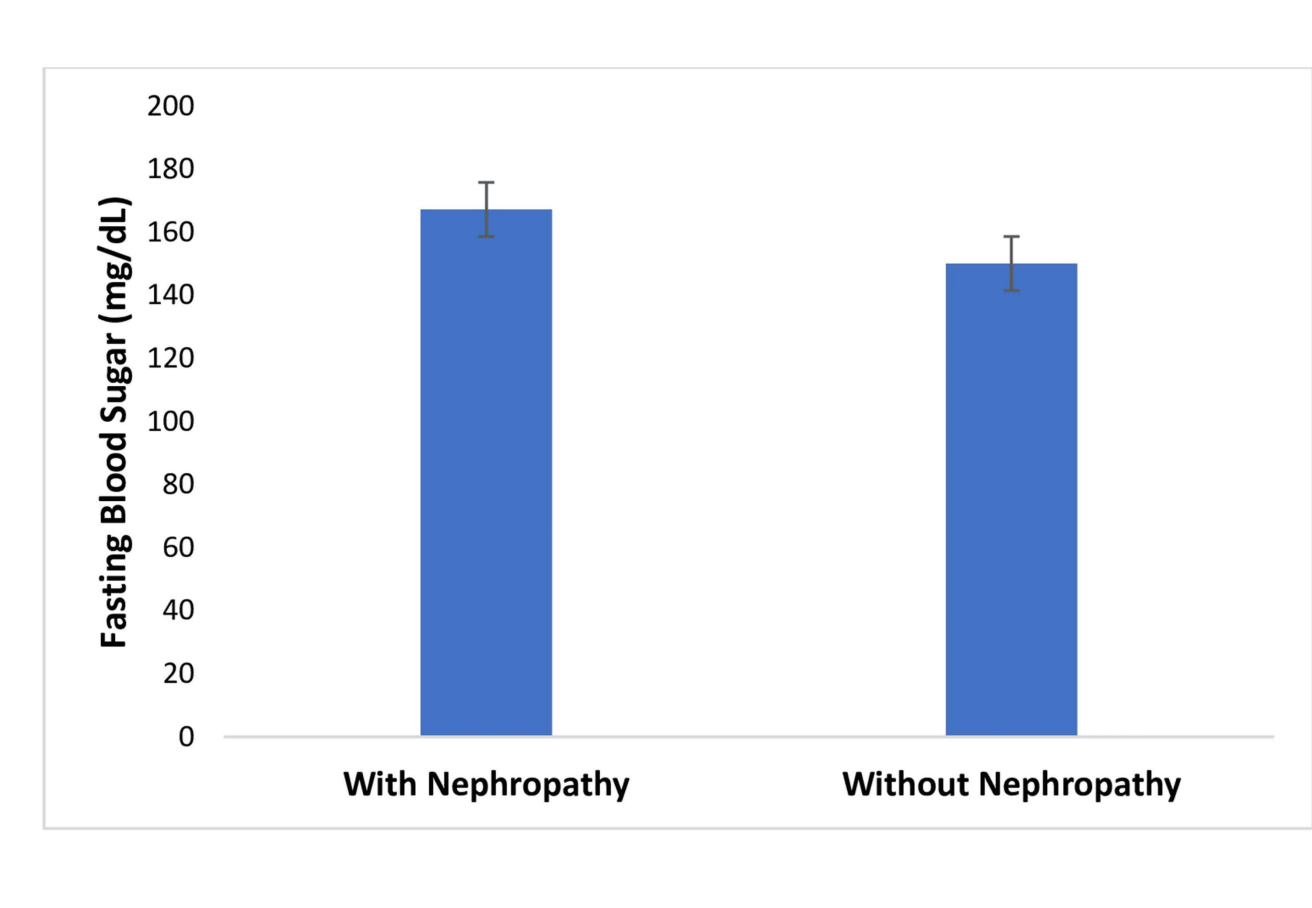
Mean fasting blood sugar of patients with diabetes.

Patients with diabetes and nephropathy have significantly higher fasting blood sugar (FBS) levels (167.22 ± 4.2 mg/dL) compared to those without nephropathy (150.07 ± 3.8 mg/dL, p = 0.03. (Figure 2).

**Figure 3:**
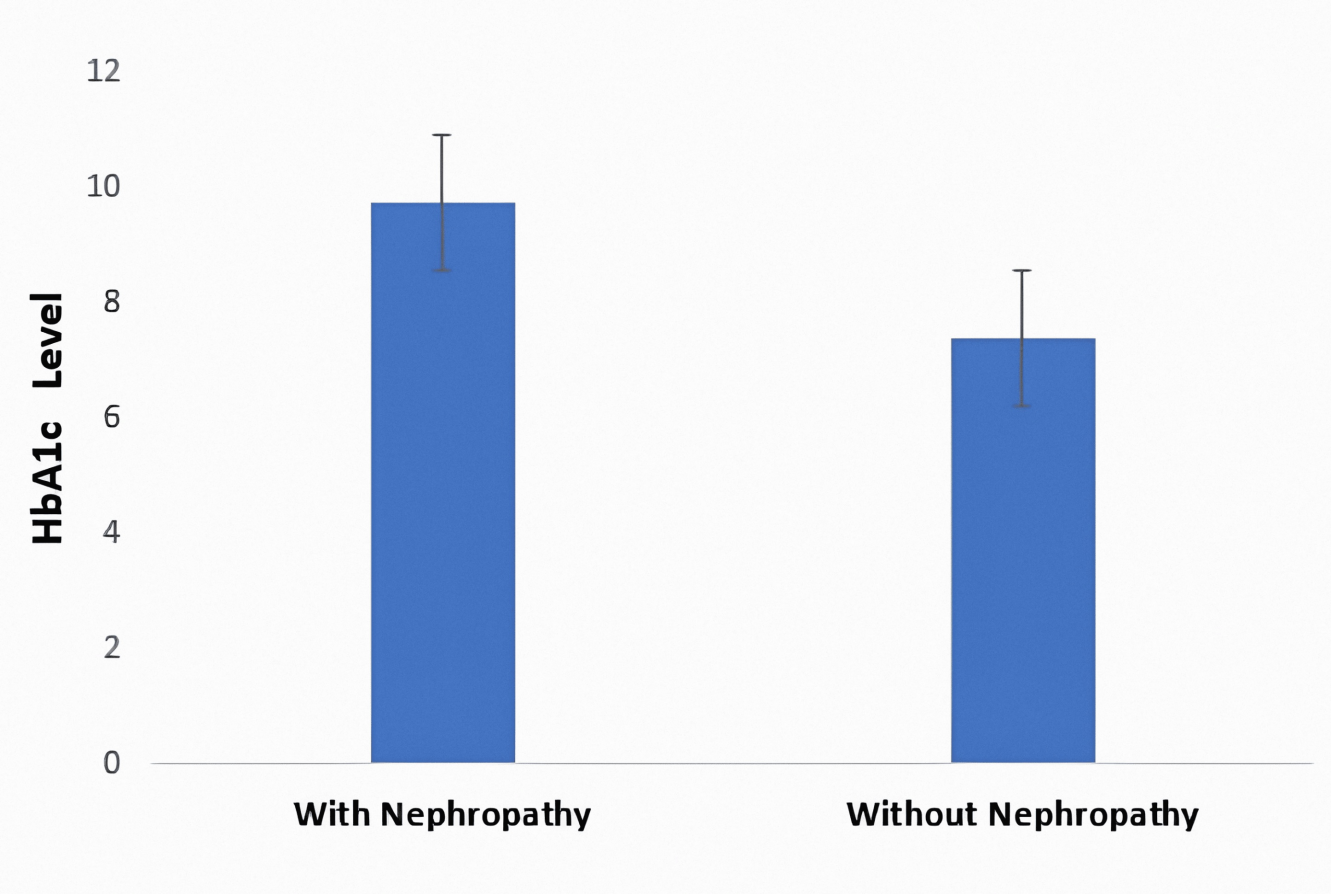
Mean HbA1c level in patients with diabetes. Bars represent mean HbA1c levels (%) in patients with and without nephropathy. Error bars indicate the standard error of the mean (SEM).

Patients with diabetes and nephropathy have significantly higher HbA1c levels (9.71 ± 1.2%) compared to those without nephropathy (7.36 ± 1.1%) (*P* = 0.02; Figure 3).

Patients with diabetes and nephropathy have higher levels of total cholesterol (197.22 mg/dL), LDL cholesterol (103.32 mg/dL), VLDL cholesterol (29.58 mg/dL), and triglycerides (145.23 mg/dL), as well as lower levels of HDL cholesterol (47.63 mg/dL), compared to those without nephropathy (Table 8).

**Table 8:** Anthropometric indices and blood pressure in patients with type 2 diabetes. Results are presented as mean ± standard deviation. *t* = independent t-test statistics used to compare the means between groups; *P* < 0.05 indicates statistical significance. HDL, high-density lipoprotein; LDL, low-density lipoprotein; VLDL, very-low-density lipoprotein

Lipid profile	With nephropathy	Without nephropathy	t	*P*-value
Total cholesterol (mg/dL)	197.22 ± 7.37	170.36 ± 8.11	9.16	0.022
HDL cholesterol (mg/dL)	47.63 ± 4.93	59.12 ± 5.67	8.81	0.030
LDL cholesterol (mg/dL)	103.32 ± 13.34	89.85 ± 11.12	8.97	0.028
HDL/LDL	0.49 ± 0.02	0.68 ± 0.01	8.94	0.011
VLDL cholesterol (mg/dL)	29.58 ± 8.46	24.06 ± 3.22	9.02	0.016
Triglyceride (mg/dL)	145.23 ± 11.31	123.23 ± 9.95	9.14	0.001

## Discussion

Diabetes mellitus is a global health concern, with increasing prevalence in both developed and developing countries [1]. Among its numerous complications, diabetic nephropathy, or kidney disease, poses a significant threat to affected individuals, leading to increased morbidity and mortality rates [4]. Understanding the prevalence and associated factors of clinical nephropathy in patients with diabetes is crucial for effective management and prevention strategies. This study investigates the prevalence and correlates of clinical nephropathy among patients with T2DM at Abia State Specialist Hospital and Diagnostic Center, Umuahia, Nigeria.

The demographic characteristics of the 255 study participants provide valuable insights into the population affected by diabetic nephropathy. The age distribution reveals that the majority of respondents were within the 40-49 (86, 33.73%) and 50-59 (74, 29.02%) age brackets, followed by those aged 60 and above (56, 21.96%). This age trend aligns with existing literature indicating that the risk of nephropathy increases with advancing age due to cumulative glycemic burden and declining renal function over time [8]. The minimal representation of younger individuals under 30 years (8.23%) further supports the view that diabetic complications tend to manifest more prominently in middle-aged and older adults. Gender distribution shows a higher prevalence of females (163, 63.92%) compared to males (92, 36.08%). While some studies report a higher prevalence of nephropathy among males due to hormonal and hemodynamic differences, others suggest that gender disparities may vary by geographic location, healthcare access, and cultural factors, which may explain the predominance of females in this study cohort [9]. Furthermore, the higher female participation could reflect better health-seeking behavior among women, a trend observed in various low- and middle-income countries [10]. Educational attainment shows that the majority of participants had secondary (109, 42.75%) or tertiary education (68, 26.67%). Only 24 (9.41%) had no formal education. Higher education levels are often associated with better health literacy and management of chronic conditions, yet this study’s findings may indicate that even among educated individuals, the risk of nephropathy remains significant, potentially due to lifestyle factors, delayed diagnosis, or insufficient health system support. Marital status data indicate that 200 (78.43%) participants were married. The role of marital status in health outcomes has been debated, with some evidence suggesting that married individuals may have better disease management due to social support [11]. However, this variable may also be influenced by age and cultural context, especially in societies where marriage is near-universal by middle age. Overall, the demographic profile observed aligns with global trends of diabetic nephropathy predominantly affecting older, married adults, with a slight variation in gender distribution. These insights reinforce the need for age- and gender-sensitive screening strategies and targeted education campaigns to improve early detection and management outcomes.

The findings of this study offer insight into the clinical profile and comorbid conditions associated with T2DM and nephropathy. A majority of participants (116, 45.49%) had been living with diabetes for 5-10 years, and 65 (25.49%) for more than 10 years, which aligns with evidence that the duration of diabetes is a strong predictor of nephropathy risk due to cumulative vascular and metabolic damage [12]. The progression of nephropathy over time reinforces the importance of early detection and continuous monitoring in primary care settings. All respondents were receiving diabetes treatment, with the most common methods being diet and exercise (138, 45.25%), insulin therapy (86, 28.20%), and oral medications (59, 19.34%). These results are consistent with the American Diabetes Association's (2023) emphasis on individualized treatment plans incorporating both pharmacologic and lifestyle approaches [13]. The diverse combination of treatment modalities highlights the importance of tailoring care to patient needs and disease severity.

Clinical nephropathy was reported in 77 (30.20%) participants, which is consistent with previous research demonstrating a high prevalence of nephropathy among diabetic populations and comparable to the prevalence reported in other low- and middle-income settings [2,8]. Similarly, a meta-analysis by Yang et al. reported a pooled prevalence of diabetic nephropathy of 35.7% among patients with T2DM worldwide [14]. These findings underscore the significance of diabetic nephropathy as a common complication of diabetes. Among these individuals, most had been diagnosed within the past five years, indicating relatively recent disease onset or improved detection rates. Although all nephropathy patients were on treatment, only 5.49% had undergone dialysis or kidney transplantation. This may reflect limited access to tertiary care or late-stage intervention, emphasizing the need for strengthened referral systems and resource allocation.

The burden of comorbidities was substantial: 194 (54.49%) had hypertension, 104 (29.21%) were obese, and 27 (7.58%) had heart disease. These findings align with global data highlighting the co-occurrence of cardiometabolic risk factors among diabetic populations [15]. Hypertension, in particular, is known to accelerate the progression of kidney disease, warranting aggressive control alongside glycemic management. A notably high proportion of participants (212, 83.14%) reported a family history of diabetes, which supports prior research on the genetic and lifestyle contributions to T2DM susceptibility [16]. This highlights the need for family-centered health education, screening, and prevention strategies. Collectively, these findings underscore the complexity of managing diabetes and its complications. They call for integrated care models that address glycemic control, comorbidity management, and early nephropathy screening, particularly for patients with long disease duration and a positive family history.

The lifestyle behaviors observed in this study provide critical insights into modifiable risk factors associated with T2DM and its complications, particularly nephropathy. Only 25 (9.8%) respondents reported smoking tobacco products, and 32 (12.55%) reported regular alcohol consumption. While these rates are relatively low, it is important to consider that even minimal exposure to smoking is associated with increased risks of cardiovascular and renal complications in individuals with diabetes [17]. Similarly, chronic alcohol use can exacerbate insulin resistance and increase the likelihood of poor glycemic control [18]. The high rate of abstention may reflect cultural norms or increased health awareness among this cohort, though underreporting remains a possibility. The majority of participants reported engaging in physical activity often (106, 41.57%) or always (87, 34.12%). Additionally, 184 (72.16%) classified their activity level as *light*, while only 42 (16.47%) engaged in *moderate* activity, and none reported *vigorous* activity. Regular physical activity is widely recognized as a protective factor against both diabetes onset and progression, including renal complications [19,20]. However, the predominance of light activity may limit metabolic benefits, particularly in populations already managing chronic conditions. Encouraging gradual progression toward moderate activity could provide greater improvements in glycemic control and kidney health. A large proportion of participants (175, 68.63%) reported following a balanced diet, while 44 (17.25%) and 29 (11.37%) adhered to high-carbohydrate and high-fat diets, respectively. While a balanced diet is ideal, the significant proportion of individuals consuming carbohydrate-heavy or fatty meals is concerning, given the association of such diets with poor glycemic control and increased cardiovascular risk [21]. Dietary interventions tailored to local food preferences and economic realities are essential for sustainable lifestyle changes.

Only 34 (13.33%) respondents reported visiting a healthcare provider regularly (at least monthly) for diabetes management, while the majority (134, 52.55%) attended occasionally (every 3-6 months), and 87 (34.12%) visited rarely (once a year or less). Regular contact with healthcare providers is essential for early detection of diabetes complications such as nephropathy [22]. Infrequent follow-up may limit opportunities for monitoring renal function and adjusting treatment regimens. A significant proportion of participants (174, 68.24%) reported encountering barriers to healthcare access, including distance and cost. These findings echo global challenges in chronic disease management in low- and middle-income countries, where geographic and financial constraints limit timely access to care [23]. Such barriers may explain the high number of participants with undiagnosed or late-stage nephropathy. Levels of knowledge about diabetic kidney disease (DKD) were generally low: only 24 (9.41%) considered themselves very knowledgeable, and 83 (32.55%) reported not being knowledgeable at all. In addition, just 41 (16.08%) had received formal education or counseling regarding DKD. These figures are concerning, as patient awareness is crucial for self-management and early symptom recognition [24]. The lack of structured education likely contributes to under-recognition and late treatment initiation. The findings highlight significant gaps in both healthcare access and patient knowledge regarding DKD, which could contribute to late diagnosis and suboptimal management outcomes.

Patients with nephropathy had a significantly higher mean BMI compared to those without nephropathy. Elevated BMI is a well-established risk factor for the development and progression of diabetic nephropathy, primarily due to its association with insulin resistance, glomerular hyperfiltration, and systemic inflammation [29]. Systolic and diastolic BPs were significantly higher in the nephropathy group. Hypertension is a known contributor to kidney damage and accelerates the progression of diabetic nephropathy through mechanisms such as increased glomerular pressure and endothelial dysfunction [30]. The elevated BP levels in this subgroup suggest a dual burden of poor glycemic and hypertensive control. Although patients with nephropathy had slightly higher average height and weight, these differences were not statistically significant. These findings imply that BMI, a derived index adjusting weight to height, accurately reflects the adiposity-related risk in this population. These observations are consistent with existing literature indicating that higher BMI and elevated BP are critical modifiable factors associated with the onset and severity of diabetic nephropathy [31]. The findings underscore the importance of integrated management strategies targeting weight control and BP reduction in diabetic care programs.

Renal function parameters revealed significant differences between patients with diabetes with nephropathy and those without, highlighting the physiological burden of kidney involvement in diabetes. Patients with nephropathy had markedly elevated levels of urea, creatinine, and uric acid, alongside a significantly reduced eGFR compared to their counterparts without nephropathy. These findings indicate clear signs of renal dysfunction, as elevated serum urea and creatinine are classic markers of impaired kidney filtration, while reduced eGFR reflects a decline in overall renal function [32]. Increased uric acid levels may also exacerbate renal damage through endothelial dysfunction and intrarenal inflammation [33]. This renal profile is consistent with the progressive nature of diabetic nephropathy, where hyperglycemia and associated metabolic abnormalities contribute to glomerular injury, eventually leading to decreased clearance of nitrogenous waste products. The significantly lower eGFR in patients with nephropathy underscores the need for early detection, renal-protective interventions, and strict glycemic and BP control to delay progression to end-stage renal disease.

The distribution of urine albumin levels among patients with diabetes revealed a predominant presence of microalbuminuria, with 156 (61.18%) falling into this category. The presence of microalbuminuria is a critical early indicator of diabetic nephropathy, reflecting the onset of glomerular injury and increased permeability to albumin. This finding aligns with the pathophysiology of DKD, where early-stage kidney damage is often characterized by the leakage of small amounts of albumin into the urine [34]. Microalbuminuria has long been recognized as a marker for both renal and cardiovascular risk in patients with diabetes, and its detection facilitates early intervention to prevent the progression to macroalbuminuria and eventual end-stage renal disease [35]. Given that 156 (61.18%) participants presented with microalbuminuria, this suggests a high prevalence of early renal involvement in this cohort, emphasizing the need for routine screening and timely management strategies to mitigate kidney damage.

The study categorized patients into different stages of clinical nephropathy based on eGFR values, with Stage 1 nephropathy being the most prevalent (40.39%). The high percentage of individuals in Stage 1 is indicative of the potential for early diagnosis and intervention before irreversible kidney damage occurs. This distribution aligns with existing literature, which emphasizes that diabetic nephropathy often begins with subtle renal changes such as hyperfiltration and microalbuminuria before progressing to more severe stages [36]. The low proportion of patients in advanced stages may reflect either successful early management or underdiagnosis in earlier stages due to a lack of awareness or routine screening. Nonetheless, this trend underscores the critical window of opportunity for implementing strategies such as glycemic control, BP management, and lifestyle modification to slow disease progression and prevent ESRD [13].

Patients with diabetes and nephropathy exhibited significantly higher FBS and HbA1c levels compared to those without nephropathy, indicating suboptimal glycemic control in this subgroup. Chronic hyperglycemia is a key driver of microvascular complications, including diabetic nephropathy, as it leads to glomerular hyperfiltration, thickening of the glomerular basement membrane, and progressive renal damage [12]. These findings are consistent with prior studies that have shown a strong association between poor glycemic control and increased risk of kidney disease in individuals with T2DM [8]. The elevated FBS levels in nephropathic patients underscore the need for intensive glucose monitoring and individualized treatment strategies aimed at maintaining blood glucose within recommended targets to slow the progression of renal impairment. Elevated HbA1c levels are a well-established risk factor for microvascular complications, including nephropathy, as prolonged hyperglycemia induces glomerular damage through mechanisms such as oxidative stress, advanced glycation end products, and inflammatory pathways [37]. The strong association between poor glycemic control and kidney damage reinforces the need for aggressive HbA1c management in patients with diabetes, especially those at risk of or already showing signs of renal impairment. Clinical guidelines recommend maintaining HbA1c levels below 7% to minimize the risk of complications, a target that appears unmet in the nephropathy group in this study [8]. This finding underscores poor glycemic control among individuals with kidney involvement and highlights the critical role of sustained hyperglycemia in the progression of diabetic nephropathy. 

The lipid profile analysis reveals significant differences between patients with diabetes and nephropathy and those without. Patients with nephropathy exhibited higher levels of total cholesterol, LDL cholesterol, VLDL cholesterol, and triglycerides, along with significantly lower HDL cholesterol and a reduced HDL/LDL ratio. These abnormalities suggest a more atherogenic lipid pattern in patients with nephropathy, which may contribute to accelerated vascular damage and progression of kidney disease. Dyslipidemia is recognized as both a consequence and a contributing factor in the pathogenesis of diabetic nephropathy, primarily through endothelial dysfunction, oxidative stress, and lipid-mediated inflammation [38]. These findings underscore the importance of regular lipid monitoring and management in patients with diabetes, especially those with renal complications, to reduce the risk of cardiovascular events and slow the progression of nephropathy.

Recommendations

Strengthen Early Screening and Diagnosis Programs

A high percentage of participants were identified at Stage 1 nephropathy, indicating a crucial window for early intervention. Regular monitoring of renal function should be part of routine diabetes care to detect nephropathy at an early stage.

Implement Aggressive Glycemic Control Measures

Significantly higher HbA1c and FBS levels in nephropathy patients underscore poor glycemic control. Intensifying blood glucose management through individualized medication plans, dietary education, and frequent monitoring is vital to slow renal disease progression [13].

Integrate Lipid Management into Diabetes Care

The study found a more atherogenic lipid profile among patients with nephropathy, including elevated total cholesterol, LDL, VLDL, and triglycerides, and lower HDL. Statins and dietary modifications should be done to reduce cardiovascular and renal risks.

*Promote BP* *Control*

Participants with nephropathy had significantly higher BPs. Clinicians should prioritize BP control with antihypertensives like ACE inhibitors or ARBs, known to confer renal protection in patients with diabetes [30].

Enhance Patient Education and Awareness

The majority of participants reported low levels of knowledge about DKD, and over 80% had never received formal education on the subject. Targeted health education programs should be integrated into clinic visits and community outreach.

Address Lifestyle and Behavioral Risk Factors

Although most participants reported engaging in light to moderate physical activity and balanced diets, some still had sedentary lifestyles, consumed alcohol, or smoked. Lifestyle counseling, exercise programs, and behavioral support can improve healthy habits while reducing comorbid risks.

Improve Access to Care and Reduce Barriers

More than two-thirds of participants faced barriers to accessing healthcare (e.g., cost, distance). Efforts should focus on improving healthcare accessibility.

Promote Family-Based Diabetes Screening and Support

The majority of participants had immediate family members with diabetes, highlighting a need for family-centered interventions. Education, early screening, and shared lifestyle goals can help prevent diabetes and its complications.

Limitations

Despite our efforts to give a reliable examination of our study objectives, our evaluation has limitations. Since the study design is cross-sectional, it only provides a snapshot in time. This limits our ability to infer causal relationships between diabetes duration, nephropathy progression, and other risk factors. Variables like alcohol use, smoking, physical activity, and knowledge about kidney disease rely on self-reported information, which is prone to recall bias and social desirability bias. Using a single center could reduce the generalizability of the findings. In the future, increasing the sample size for the power of analysis will be helpful to have more generalizable results [39].

## Conclusions

The study highlights a considerable prevalence of clinical nephropathy among patients with T2DM at Abia State Specialist Hospital and Diagnostic Center, Umuahia, Nigeria. Factors such as anthropometric indices, blood pressure, renal parameters, glycemic control, and lipid profiles significantly correlate with nephropathy presence. These findings underscore the importance of early detection and comprehensive management of nephropathy in patients with diabetes to mitigate its adverse effects. Public health interventions such as patient education, early screening, glycemic control, blood pressure management, lipid profile optimization, and comprehensive management strategies targeting modifiable risk factors are warranted to reduce the burden of nephropathy in this population. Integrating multidisciplinary approaches, improving healthcare accessibility, and addressing lifestyle behaviors are critical to mitigating the progression of diabetic nephropathy and reducing its associated morbidity. Further research, particularly longitudinal studies and intervention-based approaches, is recommended to better understand causal relationships and develop effective strategies for reducing the burden of nephropathy.

## Appendices

Appendix 

**Table 9:** Questionnaire: Prevalence and Correlates of Clinical Nephropathy in Type 2 Diabetic Patients in Abia State Specialist Hospital and Diagnostic Center, Umuahia, Nigeria: A Cross-Sectional Study Credits: Okeji IE, Uzor OF, Okafor CC, Okafor SU, Airaodion AI, Orji OJ, Abali IO, Esangbedo IJ, Jolaoye OO, Olaotan TO, Zacs IC, Umeh AB, Ojumonu UE, Ekwere AM, Madueke CK, Austin-Jemifor O, Victor-Anozie EN, Amuta AC, Umeh C.

Section	Question No.	Question	Response Options
Informed Consent	–	You are invited to participate in a research study titled Prevalence and Correlates of Clinical Nephropathy... Participation is voluntary, responses are confidential, and you may withdraw at any time.	☐ I have read and understood the information above and voluntarily agree to participate.
Sociodemographic Info	1	Age	☐ Below 20 ☐ 20–29 ☐ 30–39 ☐ 40–49 ☐ 50–59 ☐ 60 and above
	2	Sex	☐ Male ☐ Female
	3	Educational Level	☐ No formal education ☐ Primary ☐ Secondary ☐ Tertiary
	4	Marital Status	☐ Single ☐ Married ☐ Divorced/Widowed
Clinical Information	5	Duration with T2DM	☐ <1 year ☐ 1–3 yrs ☐ 3–5 yrs ☐ 5–10 yrs ☐ >10 yrs
	6	Currently receiving diabetes treatment?	☐ Yes ☐ No
	7	Type(s) of diabetes treatment (select all that apply)	☐ Oral meds ☐ Insulin ☐ Diet/exercise ☐ Other: _________
	8	Diagnosed with clinical nephropathy (kidney disease)?	☐ Yes ☐ No
	9	Duration with nephropathy	☐ <1 year ☐ 1–3 yrs ☐ 3–5 yrs ☐ 5–10 yrs ☐ >10 yrs
	10	Receiving treatment for nephropathy?	☐ Yes ☐ No ☐ Not applicable
	11	Ever undergone dialysis or kidney transplant?	☐ Yes ☐ No
	12	Other chronic conditions (select all that apply)	☐ Hypertension ☐ Heart disease ☐ Obesity ☐ Other: _______ ☐ Not applicable
Lifestyle And Behavioural Factors	13	Any immediate family with diabetes?	☐ Yes ☐ No
	14	Smoke tobacco products?	☐ Yes ☐ No
	15	Consume alcohol regularly?	☐ Yes ☐ No
	16	Frequency of physical activity?	☐ Always ☐ Often ☐ Sometimes ☐ Rarely ☐ Never
	17	Level of physical activity:	☐ Sedentary ☐ Light ☐ Moderate ☐ Vigorous
	18	Dietary pattern:	☐ High fat ☐ High carbohydrate ☐ Balanced ☐ Other: ____________
Access To Healthcare And Knowledge	19	Frequency of healthcare visits for diabetes management:	☐ Regularly ☐ Occasionally ☐ Rarely
	20	Encountered barriers to healthcare access?	☐ Yes ☐ No
	21	Knowledge about diabetic kidney disease:	☐ Very ☐ Somewhat ☐ Not very ☐ Not at all
	22	Received formal education/counselling on diabetic kidney disease?	☐ Yes ☐ No
Conclusion	Thank you for taking the time to complete this questionnaire. Your responses will help health professionals and researchers better understand how to address diabetic kidney disease among Type 2 diabetic patients in Nigeria.
☐ I confirm that I have answered this questionnaire truthfully and to the best of my knowledge.
